# Identification of Novel Gene-Specific Markers for Differentiating Various Pathogenic *Campylobacter* Species Using a Pangenome Analysis Approach

**DOI:** 10.3390/pathogens14050477

**Published:** 2025-05-14

**Authors:** Emmanuel Kuufire, Kingsley E. Bentum, Rejoice Nyarku, Viona Osei, Asmaa Elrefaey, Tyric James, Yilkal Woube, Raphael Folitse, Temesgen Samuel, Woubit Abebe

**Affiliations:** 1 Center for Food Animal Health, Food Safety and Defense, Department of Pathobiology, College of Veterinary Medicine, Tuskegee University, Tuskegee, AL 36088, USA; ekuufire9436@tuskegee.edu (E.K.); kbentum8786@tuskegee.edu (K.E.B.); rnyarku8794@tuskegee.edu (R.N.); vosei3882@tuskegee.edu (V.O.); aelrefaey3754@tuskegee.edu (A.E.); tjames2981@tuskegee.edu (T.J.); ywoube@tuskegee.edu (Y.W.); tsamuel@tuskegee.edu (T.S.); 2 School of Veterinary Medicine, Kwame Nkrumah University of Science and Technology, Kumasi AK-385-1973, Ghana; raphfolitse@yahoo.com

**Keywords:** *Campylobacter* spp., pangenome analysis, species-specific markers, molecular detection, foodborne pathogens

## Abstract

*Campylobacter* spp. are the causative agents of campylobacteriosis, a major foodborne illness globally, with millions of cases reported annually. These pathogens pose significant risks to both human and animal health. Conventional culture-based diagnostic methods are labor-intensive and time-consuming, underscoring the need for more efficient molecular detection strategies. This study employed a pangenomic analysis to identify novel gene-specific markers for pathogenic *Campylobacter* species and subspecies, laying the groundwork for their application in diverse diagnostic assays. A curated dataset of 105 high-quality genomes, representing 33 species and 9 subspecies, was analyzed using the Roary ILP Bacterial Annotation Pipeline. The results revealed substantial genomic diversity within the genus, with core gene counts varying across different nucleotide identity thresholds. Ribosomal genes such as *rpsL*, *rpsJ*, *rpsS*, *rpmA*, *rpsK*, *rpsU*, *rpsG*, *rpmH*, and *rpsZ* were consistently identified in the core genome, whereas accessory genes exhibited marked variability. This study uncovered novel and highly specific genetic markers for various *Campylobacter* species, including *petB*, *clpX*, and *carB* for *C. coli*; hypothetical proteins for *C. jejuni* and *C. fetus*; *porA2* for *C. lari*; and *mdtJ* for *C. upsaliensis*. These markers demonstrated a specificity of at least 90% with minimal cross-reactivity with non-target organisms. The findings underscore the genomic heterogeneity within *Campylobacter* and provide essential genetic targets for the enhanced molecular detection of its pathogenic species, subspecies, and biovars.

## 1. Introduction

Foodborne bacterial enteric diseases continue to pose a significant global health concern, causing an estimated 2.2 million deaths annually and contributing to the loss of approximately 112,000 disability-adjusted life years in the United States alone [1,2,3]. Among these pathogens, *Campylobacter* species are recognized as leading agents of foodborne illnesses, raising serious public health concerns both in the United States [4] and globally [5]. According to the Centers for Disease Control and Prevention, there are an estimated 1.5 million *Campylobacter* infections each year in the United States, with associated economic costs ranging from USD 1.3 billion to USD 6.8 billion [6,7]. Recent statistics report an incidence rate of approximately 20 infections per 100,000 population [8].

*Campylobacter* infections occur more frequently than those caused by *Salmonella* or *Escherichia coli*, with *Campylobacter* species isolated from patients with gastrointestinal infections at rates 3–4 times higher than other notable enteric pathogens [9]. They account for over 60% of all reported zoonotic cases [10].

Infection sources include raw milk [11], undercooked poultry [12], contaminated fruit and vegetables [13,14], and fecal–oral transmission routes, such as ingestion of contaminated water [15,16] or exposure in environments like children’s playgrounds [17]. A recent attribution study has identified poultry as a primary reservoir of human *Campylobacter* infections in the United States, accounting for an estimated 68% of clinical isolates, followed by cattle, wild birds, and pork [18].

Of the 23 known pathogenic species [19,20], *C. jejuni* and *C. coli* are the primary causes of *Campylobacter* infections, with *C. jejuni* alone responsible for up to 90% of reported human cases [11,21]. Infection with *C. jejuni* is particularly concerning due to its potential to trigger an autoimmune disorder. This is attributed to molecular mimicry between the bacterium’s outer membrane components and human nerve cell structures, which can result in nerve damage [22]. Other species such as *C. concisus* have been associated with inflammatory bowel diseases, including Crohn’s disease [23,24]. Although species like *C. lari*, *C. upsaliensis*, and *C. fetus* are less frequently implicated in human infections, they nonetheless contribute to the overall public health burden [25]. Severe complications are most commonly observed in immunocompromised individuals [26].

Beyond their impact on human health, *Campylobacter* species have also significantly affected animal health, especially in livestock. *Campylobacter fetus* subsp. *venerealis* is the main cause of bovine genital campylobacteriosis, a sexually transmitted disease that leads to infertility and embryonic death in cattle [27,28]. Similarly, *C. fetus* subsp. *fetus* has long been linked to epizootic abortions in sheep and goats, causing metritis and placentitis characterized by hemorrhagic, necrotic cotyledons. This highlights the pathogen’s ability to infect both digestive and reproductive systems [29]. More recently, *C. jejuni* has become the leading cause of ovine abortions in the United States, driven by the emergence of a hypervirulent, tetracycline-resistant strain [27].

Despite the significant public health threat posed by *Campylobacter*, conventional culture-based detection methods remain time-consuming and labor-intensive, requiring microaerophilic conditions and often taking several days to produce results [30]. These limitations highlight the urgent need for faster and more reliable detection methods. Culture sensitivity is particularly low for non-*C. jejuni/coli* species, resulting in underreporting and misdiagnosis [31]. To address these challenges, advanced molecular and proteomic detection techniques have gained prominence. Polymerase chain reaction (PCR)-based assays allow for faster and more sensitive identification of *Campylobacter DNA* in clinical, food, and environmental samples [32]. Matrix-assisted laser desorption ionization–time of flight mass spectrometry (MALDI-TOF) has enhanced the species-level characterization of cultured isolates [33], though it still relies on successful cultivation and cannot differentiate closely related strains. While PCR remains a valuable tool for pathogen detection, its effectiveness is hindered by inhibitors in complex sample matrices and the need for enrichment steps to improve sensitivity [32].

Recent advances have leveraged the genetic variability present within *Campylobacter* species, particularly among pathogenic strains [20]. However, the frequent horizontal exchange of genetic material among diverse *Campylobacter* species and strains further complicates accurate detection [34]. Consequently, the identification of highly specific genetic targets within *Campylobacter*, especially pathogenic species and subspecies, remains crucial for improving detection accuracy. Accurate species-level identification of *Campylobacter* is essential in clinical diagnostics, as it informs treatment decisions due to species-specific differences in antimicrobial resistance patterns [30]. Likewise, high-resolution typing plays a critical role in epidemiology and outbreak investigations by identifying sources and transmission routes, thereby supporting effective control measures [24]. In the food industry and veterinary settings, species- and subspecies-level characterization is equally important for source attribution and targeted interventions, given the distinct host reservoirs and disease outcome manifestations associated with different *Campylobacter* taxa [18]. Although large-scale genomic sequencing initiatives have heightened clinical awareness of *Campylobacter*, few studies have focused on exploring its genomic diversity and identifying species-specific targets. This study utilizes a pangenome approach to discover novel, species-specific genetic markers aimed at developing rapid, reliable, and cost-effective assays for *Campylobacter* detection. To our knowledge, this is the first investigation to directly identify markers at both the species and subspecies levels for pathogenic *Campylobacter* using integrated pangenome and core genome analyses. These findings will enhance food safety by providing gene-specific targets for detection assays.

## 2. Materials and Methods

### 2.1. Data Collection

#### Bacterial Genomes Used for the Study

A total of 132 complete reference genomes representing 33 known *Campylobacter* species and 9 subspecies [19,20] were retrieved from the National Center for Biotechnology Information (NCBI) database on 18 July 2024. The dataset includes a minimum of one and a maximum of five strains per species and subspecies. To ensure data quality, genomes were filtered using the CheckM tool (version 1.2.3) [35], applying thresholds of ≥90% completeness, ≤5% heterogeneity, and ≤5% contamination. Following this quality assessment, 105 genomes were retained for downstream analysis. The dataset includes the following *Campylobacter* species and subspecies, with the number of genomes indicated in parentheses: *Campylobacter armoricus* (2), *Campylobacter avium* (2), *Campylobacter canadensis* (1), *Campylobacter coli* (3), *Campylobacter concisus* (3), *Campylobacter corcagiensis* (2), *Campylobacter cuniculorum* (2), *Campylobacter curvus* (2), *Campylobacter devanensis* (1), *Campylobacter fetus* (2), *Campylobacter fetus* subsp. *fetus* (2), *Campylobacter fetus* subsp. *testudinum* (2), *Campylobacter fetus* subsp. *venerealis* (4), *Campylobacter gracilis* (2), *Campylobacter helveticus* (3), *Campylobacter hepaticus* (2), *Campylobacter hominis* (4), *Campylobacter hyointestinalis* subsp. *hyointestinalis* (2), *Campylobacter hyointestinalis* subsp. *lawsonii* (2), *Campylobacter iguaniorum* (3), *Campylobacter insulaenigrae* (3), *Campylobacter jejuni* (5), *Campylobacter jejuni* subsp. *doylei* (2), *Campylobacter jejuni* subsp. *jejuni* (5), *Campylobacter lanienae* (2), *Campylobacter lari* (2), *Campylobacter lari* subsp. *concheus* (2), *Campylobacter lari* subsp. *lari* (2), *Campylobacter mucosalis* (2), *Campylobacter novaezeelandiae* (1), *Campylobacter ornithocola* (1), *Campylobacter peloridis* (2), *Campylobacter pinnipediorum* subsp. *pinnipediorum* (2), *Campylobacter porcelli* (1), *Campylobacter rectus* (2), *Campylobacter showae* (4), *Campylobacter spp* (2), *Campylobacter sputorum* (1), *Campylobacter sputorum* bv. *faecalis* (1), *Campylobacter sputorum* bv. *paraureolyticus* (1), *Campylobacter sputorum* subsp. *sputorum* (2), *Campylobacter subantarcticus* (2), *Campylobacter upsaliensis* (3), *Campylobacter ureolyticus* (3), *Campylobacter vicugnae* (3), and *Campylobacter volucris* (3). Accession numbers for all selected genomes are provided in the Supplementary Data (Supplementary Table S1).

### 2.2. Data Analysis

#### 2.2.1. Pangenome Analysis to Identify Core and Accessory Genes

**Figure 1:**
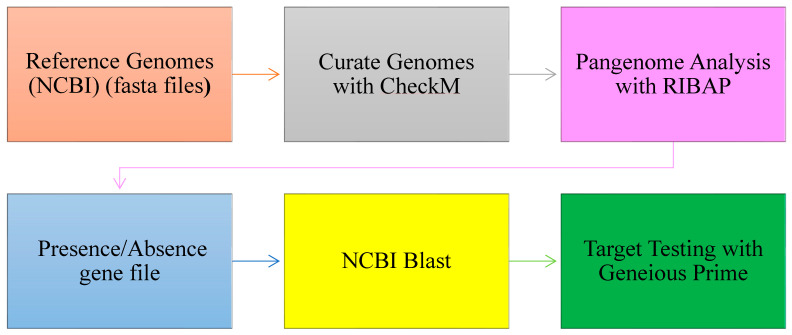
Workflow for target gene identification: from pangenome analysis to BLAST (version 2.1.2) validation and functional testing.

The FASTA genome sequences of the various species and subspecies were analyzed using the Roary ILP Bacterial Core Annotation Pipeline (RIBAP) [36], executed within the Nextflow workflow management system [37]. Briefly, genomes were first annotated using Prokka, and core genome alignment was performed using Roary [38]. The pipeline integrated an integer linear programming (ILP) module to refine gene clusters predicted by Roary, facilitating the identification of both core and accessory genes, including unique species- and subspecies-specific genes. The average nucleotide identity (ANI) thresholds applied during the RIBAP analysis were 60%, 70%, 80%, 90%, and 95% and were visualized utilizing Phandango [39]. While lower ANI thresholds aided in a broad target exploration, the 95% threshold yielded more refined and stringent results for species identification and classification. A summary of the workflow is presented in Figure 1.

#### 2.2.2. Selection of Unique Targets for *Campylobacter* spp. Detection

Conserved unique genes across *Campylobacter* species and subspecies were selected as potential gene-specific targets using the gene presence/absence matrix (present = 1, absence = 0) generated through the pangenome analysis. The accessory genome, comprising genes found only in subsets of *Campylobacter* genomes, was systematically examined to identify species- and subspecies-specific genetic markers. The genetic diversity found within the accessory genome of *Campylobacter* enables the identification of unique genetic markers that differentiate each pathogenic species and subspecies.

#### 2.2.3. In Silico Validation of the Selected Targets Across Diverse Campylobacter Strains

The uniqueness of the identified genetic markers was validated in silico using the MegaBLAST program embedded in the NCBI’s Standalone BlAST (Basic Local Alignment Search Tool) package (version 2.1.2) and Geneious Prime software (Geneious Prime^®^ 2024.0.2) (https://www.geneious.com). Genetic markers with query hits showing ≥90% sequence identity were considered strong candidates for further application.

#### 2.2.4. Functionality of Core Genes and Identified Genetic Targets

Predicted Gene Ontology (GO) terms from the UniProt database (accessed on 20 December 2024) were used to assess the molecular functions, biological processes, and predicted cellular localizations of both the core genes and the species-specific genetic targets identified in this study. We used Uniprot database release version of 2024_06.

## 3. Results

### 3.1. Genomic Diversity and Characteristics of Curated Strains of Campylobacter

Analysis of 105 high-quality *Campylobacter* genomes revealed an open pangenome, reflecting considerable genetic diversity across species and subspecies. The core genome, defined by the presence of conserved genes at varying nucleotide identity thresholds, comprised 226 genes at 60% identity, 119 at 70%, 46 at 80%, 9 at 90%, and a single gene at 95% identity, as summarized in Table 1. Core genes identified at the 90–95% identity threshold primarily included ribosomal genes such as *rpsL*, *rpsJ*, *rpsS*, *rpmA*, *rpsK*, *rpsU*, *rpsG*, *rpmH*, and *rpsZ*, along with a *soj* gene.

**Figure 2:**
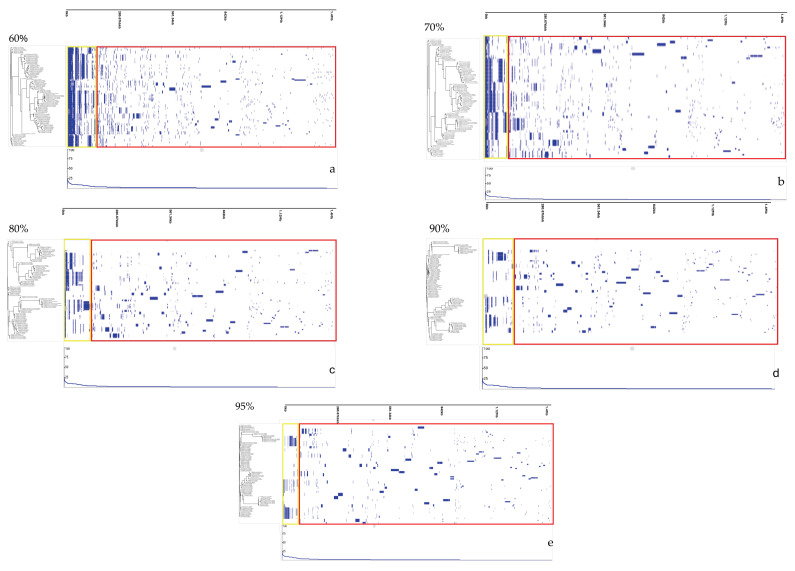
Gene presence/absence matrices at varying average nucleotide identity (ANI) thresholds, as analyzed using Phandango. Subfigures (**a**–**e**) correspond to ANI thresholds of 60%, 70%, 80%, 90%, and 95%, respectively. Yellow boxes represent core and soft-core genes, while red boxes indicate accessory genes.

**Figure 3:**
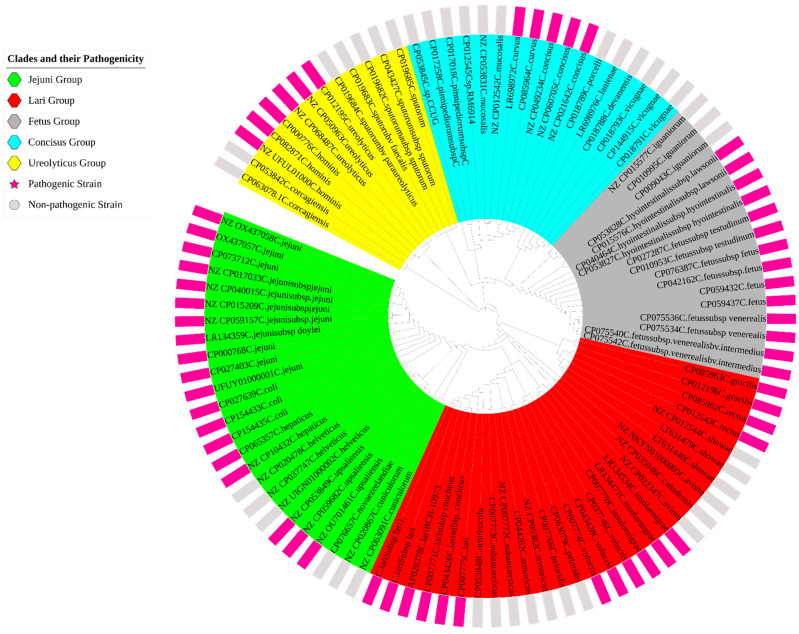
Pangenome distribution of the *Campylobacter* genus, illustrating the major clades identified within the analyzed dataset. The figure differentiates between pathogenic and non-pathogenic strains, represented by pink and gray bars, respectively.

Soft-core genes, found in most of the genomes but not universally conserved, included *rplT*, *rplK*, *rplN*, *tufA*, *fusA*, *frdB*, *rpmG2*, *rplP*, *rpsM*, *dnaK*, *nrdB*, and *cheY*. In contrast, the accessory genome showed extensive variability, ranging from 25,118 genes at the 60% identity threshold to 70,165 genes at 95%, as illustrated in Figure 2. Further analysis of the pangenome distribution revealed distinct *Campylobacter* clades, including the Jejuni, Lari, Ureolyticus, Concisus, and Fetus groups, as shown in Figure 3. These groupings represent the most pathogenic *Campylobacter* species within each phylogenetic clade, based on their virulence and epidemiological significance.

### 3.2. Specific Genetic Targets and In Silico Validation (Sensitivity and Specificity)

**Figure 4:**
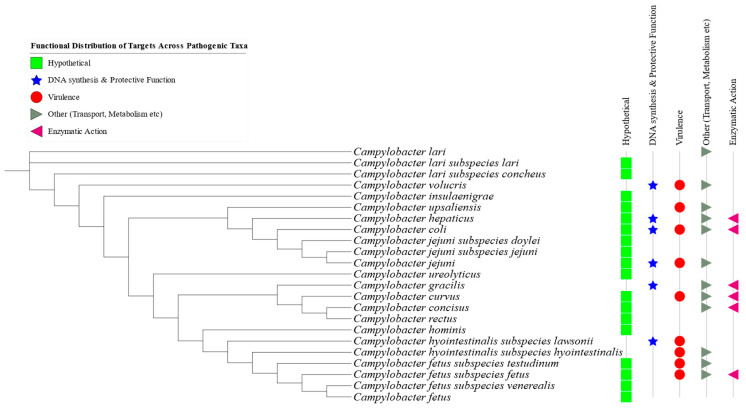
Functional distribution of the identified genetic targets across various pathogenic *Campylobacter* species and subspecies. Green rectangles represent hypothetical proteins; blue stars indicate genes involved in DNA synthesis and protective functions; red bullets denote virulence-associated genes; gray symbols represent genes related to transport and metabolism; and pink triangles indicate genes involved in enzymatic activities.

Pangenome analysis identified a total of 54 unique genetic markers across various pathogenic *Campylobacter* species and subspecies, as summarized in Table 2. At least one distinctive protein per taxon was selected based on gene presence/absence profiles (see Supplementary Data). The selected targets were validated in silico across 23 pathogenic *Campylobacter* taxa (Figure 4).

BLASTN analysis of the candidate genes revealed high specificity and coverage, with most targets achieving 100% sequence coverage and showing no cross-reactivity with non-target *Campylobacter* or other non-*Campylobacter* organisms.

For example, the altronate dehydratase protein in *C. insulaenigrae* showed 100% coverage with three hits and no cross-reactivity. Similarly, the peptidoglycan O-acetyltransferase in *C. volucris* returned three hits with 99.93% identity and less than 84.35% identity with four other *Campylobacter* species, confirming its specificity. The hypothetical protein identified in *C. lari* subsp. *concheus* had 15 unique hits without any cross-reactivity. The major outer membrane protein (*porA_2*) of *C. lari* had 62 hits, although it was also present in four other *Campylobacter* species. In contrast, the two markers for *C. lari* subsp. *lari* had two hits at 100% identity and no matches in other organisms.

The spermidine export protein target for *C. upsaliensis* yielded six hits with 99.74% identity and 100% coverage. *C. hepaticus* was associated with six specific hits for the l-asparaginase protein. For *C. coli*, all six unique targets showed 100 hits with 97.05% identity and 100% coverage, without any cross-reactivity.

For *C. jejuni* subsp. *doylei*, a hypothetical protein produced five hits at 99.13% identity. *C. jejuni* yielded 100 hits at 100% identity across two unique hypothetical proteins, with no cross-reactivity observed. *C. jejuni* subsp. *jejuni* had five specific hits, while *C. ureolyticus* had three hits for its unique hypothetical protein, also without cross-reactivity.

Targets for *C. gracilis*, *C. curvus*, *C. concisus*, *C. rectus*, and *C. hominis* all demonstrated 100% identity and high specificity, with no off-target matches. The markers for *C. hyointestinalis* subsp. *lawsonii* and *C. hyointestinalis* subsp. *hyointestinalis* returned hits at identity levels of 99.43% and above. For *C. fetus subsp. testudinum*, the lipopolysaccharide protein had four hits and seven for each of the two additional targets with a 100% identity match. The *cas1b* gene in *C. fetus* subsp. *fetus* showed 99.73% identity across five hits. Finally, *C. fetus* subsp. *veneralis* had 12 specific hits with two hypothetical proteins at 100% identity, though two additional proteins showed cross-reactivity with other *C. fetus* subspecies.

### 3.3. Functionality of Core Genes and Specific Genetic Targets

Predicted Gene Ontology (GO) terms, retrieved from the UniProt database (accessed on 20 December 2024), indicated that the annotated genes were predominantly associated with transferase, lyase, and hydrolase activity. These enzymes are integral to functions such as DNA binding, DNA repair, molecular transport, and ATP-dependent processes. In terms of biological processes, the genes were primarily involved in carbohydrate metabolism, protein catabolism, mitochondrial organization, and cellular signaling pathways and, in some cases, exhibited potential virulence-related roles (Figure 4).

The predicted cellular localization suggested that these gene products were primarily situated in the cytosol, plasma membrane, flagellum, and other subcellular compartments. Accession numbers and corresponding GO terms for the closest related proteins, identified through NCBI BLASTP searches, are provided in Table 2 and detailed further in the Supplementary Data.

Of the 54 protein targets identified, 28 were classified as hypothetical proteins with no currently known molecular functions, biological processes, or subcellular localizations (Table 2). These hypothetical proteins represent promising candidates for future functional characterization.

## 4. Discussion

This study comprehensively investigated the pangenome of the *Campylobacter* genus, identified species-specific genetic markers, and offered functional insights into these markers. The findings underscore several important aspects of *Campylobacter* biology, including its pronounced genomic heterogeneity, species and subspecies diversity, and the potential for achieving highly specific molecular detection based on the markers identified.

The analysis revealed an open pangenome characterized by a markedly reduced core genome and a vast number of accessory genes, illustrating the substantial genetic flexibility and adaptability of the genus. These observations are consistent with previous findings [40,41]. By classifying the pangenome into core, soft-core, and accessory genes, this study further highlights the evolutionary plasticity of *Campylobacter* [42], which appears to exhibit more rapid genome evolution than other enteric pathogens such as *Escherichia coli* and *Salmonella* Typhimurium [43].

A total of 226 core genes were identified at a 60% average nucleotide identity (ANI) threshold. This number decreased dramatically to a single gene at 95% ANI, closely aligning with the results reported by Wu et al. [19]. This steep decline in core gene counts with increasing ANI threshold reflects the considerable genetic variation within *Campylobacter* species [26], likely driven by ecological pressure and evolutionary adaptation [44].

Most core genes identified in this study were ribosomal, except for the *soj* gene. Ribosomal genes are essential for ribosomal biogenesis, structural stability, and survival under stress conditions. The *soj* gene, which encodes a sporulation initiation inhibitor protein, may play a key regulatory role in bacterial survival during environmental stress [45]. Its presence in *Campylobacter*, a genus not known to sporulate, suggests a functional parallel to its ortholog *Bacillus subtilis*, where *soj* inhibits sporulation by preventing the accumulation of the activator protein Spo0A and by directly repressing Spo0A-dependent gene expression [45]. This functional analogy may provide insight into why *Campylobacter* species lack a sporulation pathway despite the presence of regulatory elements.

Within the accessory genome, “shell genes” were found to be relatively stable and widely distributed across some *Campylobacter* genomes, whereas “cloud genes” were more dynamic and less conserved. This broad diversity in accessory gene content underscores *Campylobacter’s* remarkable genomic plasticity and its ability to adapt to various environments and host organisms [26,46]. These genes likely reflect the acquisition of novel traits related to virulence, antibiotic resistance, and host specificity [47,48].

In this study, at least one unique genetic marker was identified for each of the pathogenic *Campylobacter* species and subspecies, an approach paralleling similar work in other foodborne pathogens such as *Salmonella* [1,49,50], where pangenome strategies have also proven effective. Previous research, such as that by Zhong et al. [26], constructed pangenomes across *Campylobacter* species to reveal the breadth of genetic diversity, species-specific virulence traits, and antimicrobial resistance profiles. Wu et al. [19] proposed a standardized ANI threshold of 94.2% for delineating novel *Campylobacter* species. However, many such studies employed ANI thresholds below 95%, which may not fully resolve intra-species diversity and often do not directly identify species-specific targets.

This study diverges from the more common reference-based approach, which relies on mapping genomes to a single reference to identify variable sites such as single-nucleotide polymorphisms (SNPs) [51]. While useful for examining the conserved core genome among isolates, reference-based methods may overlook novel or unique genes and are limited in capturing the full scope of the accessory genomes [52]. In contrast, the pangenome approach used here allows for the detection of gene content variation across diverse strains and the discovery of functionally relevant targets.

The genetic markers identified in this study hold significant promise for species-level identification, which is critical for diagnostics and epidemiological surveillance [20,50]. BLASTN analyses revealed that the selected targets were highly specific to the 23 pathogenic *Campylobacter* species and subspecies, exhibiting minimal or no cross-reactivity with non-target organisms. Further validation using Geneious Prime supported the utility of these markers as robust tools for detecting *Campylobacter* in food, clinical, and environmental samples.

These markers are ideally suited for rapid, sensitive, and specific molecular detection techniques, including polymerase chain reaction (PCR) and isothermal amplification assays. This mirrors similar efforts in *Salmonella* research, where real-time PCR was used to detect serovar-specific markers [50]. Additionally, considerations such as GC content and sequence length were factored into the marker selection, ensuring optimal suitability for primer design [53] and downstream diagnostic applications.

Interestingly, 51.9% of the identified species-specific genes were annotated as hypothetical proteins, suggesting that many *Campylobacter* species harbor unique and uncharacterized functions. These uncharacterized proteins may play critical roles in pathogenicity or adaptation to specific environmental niches. Further research will be necessary to elucidate their functional roles, which could reveal novel targets for therapeutic or diagnostic development. In contrast, the remaining identified targets were predicted to be involved in key cellular metabolic processes, including carbohydrate metabolism, signaling pathways, and virulence mechanisms that may contribute to the bacterium’s ability to infect a host [26] and persist under diverse environmental conditions [20].

## 5. Conclusions

This study offers valuable insights into the genomic diversity of *Campylobacter*, its functional genetic landscape, and the potential for developing specific diagnostic markers. The pangenome analysis underscores the remarkable genetic flexibility and adaptability of *Campylobacter*, enabling its survival across a wide range of ecological niches. The identification and in silico validation of species-specific genetic markers present promising tools for the rapid molecular detection of *Campylobacter* infections in both clinical and food safety applications.

Looking ahead, experimental validation of these markers using real-world *Campylobacter* isolates will be essential to confirm their reliability and diagnostic accuracy. In addition, further investigation into the roles of the identified hypothetical proteins, particularly their potential involvement in pathogenicity, stress response, and host adaptation, is recommended. These studies could lead to the discovery of novel therapeutic and vaccine targets to combat *Campylobacter* infections and contamination, especially as the pathogen continues to evolve.

## Figures and Tables

**Table 1:** Distribution of core, soft-core, shell, and cloud genes across varying average nucleotide identity (ANI) thresholds.

Classification of Genes		60%	70%	80%	90%	95%
Core genes		226	119	46	9	1
Soft-core genes		77	49	23	3	1
Accessory genes	Shell genes	2419	2529	2031	889	339
	Cloud genes	22,699	33,788	43,063	53,319	69,826

**Table 2:** Identified genetic targets across pathogenic *Campylobacter* species and subspecies, with corresponding accession numbers and sequence lengths.

Pathogenic Species/Subspecies	Protein Name (Gene)	Accession No.	Size (bp)
*C. coli*	Cytochrome b (*PetB*) **	WP_038836333.1	1248
*C. coli*	ATP-binding subunit (*ClpX*) **	WP_002778039.1	1224
*C. coli*	ATP-dependent Clp protease **	WP_264378315.1	291
*C. coli*	Transaldolase **	WP_289867517	987
*C. coli*	Hypothetical protein **	WP_002779418.1	171
*C. coli*	Carbamoyl-phosphate synthase large subunit (*carB*) **	WP_264378315.1	3270
*C. concisus*	Hypothetical protein **	WP_072594306.1	432
*C. concisus*	Hypothetical protein	WP_103643789.1	429
*C. concisus*	Phosphoribosylformlyglcinsmidine cyclo-ligase	WP_107892589.1	984
*C. curvus*	Hypothetical protein **	WP_009649311.1	711
*C. curvus*	Hypothetical protein **	WP_018136234.1	696
*C. curvus*	Virulence protein **	WP_011992574.1	393
*C. curvus*	Bifunctional enzyme IspD/IspF (*ispDF*)	WP_018136231.1	1116
*C. fetus*	Hypothetical protein **	WP_024305373.1	1296
*C. fetus*	Hypothetical protein **	WP_144685876.1	654
*C. fetus* subp. *testudinum*	Lipopolysaccharide export system protein (*lptA*)	WP_023385482.1	468
*C. fetus* subp. *testudinum*	Hypothetical protein	WP_039362567.1	1248
*C. fetus* subp. *testudinum*	Hypothetical protein	WP_058909030.1	936
*C. fetus* subsp. *fetus*	Type I-B CRISPR associated endonuclease Cas1 (*cas1b*)	WP_041738340.1	1002
*C. fetus* subsp. *fetus*	Hypothetical protein	WP_038454040.1	228
*C. fetus* subsp. *veneralis*	Hypothetical protein **	WP_303297428.1	243
*C. fetus* subsp. *veneralis*	Hypothetical protein **	WP_303297427.1	297
*C. fetus* subsp. *veneralis*	Hypothetical protein	WP_002850340.1	261
*C. fetus* subsp. *veneralis*	Hypothetical protein	AIR80954.1	279
*C. gracilis*	Putative oxidoreductase	WP_050346355.1	855
*C. gracilis*	DNA adenine methylase **	EEV17345.1	813
*C. gracilis*	Apolipoprotein N-acyltransferase **	WP_005872283.1	1182
*C. hepaticus*	Hypothetical protein	WP_124134096.1	780
*C. hepaticus*	L-asparaginase 2 (*ansA*) **	MDX2324043.1	231
*C. hominis*	Hypothetical protein **	WP_012109050.1	711
*C. hominis*	Hypothetical protein **	WP_011991502.1	918
*C. hyointestinalis* subsp. *hyointestinalis*	Arginine exporter protein (*argO*)	WP_232051094.1	603
*C. hyointestinalis* subsp. *lawsonii*	Beta sliding clamp	WP_063997406.1	1071
*C. hyointestinalis* subsp. *lawsonii*	Manganese transport system membrane protein (*mtB_3*)	WP_151062156.1	825
*C. hyointestinalis* subsp. *lawsonii*	Flagellar biosynthesis protein (*flhA*)	WP_244948766.1	2160
*C. insulaenigrae*	Altronate dehydratase (*uxaA_2*) **	WP_039651237.1	264
*C. insulaenigrae*	Flavin-dependent thymidylate synthase (*thyX*)	WP_039648794.1	633
*C. insulaenigrae*	4-hydroxy-tetrahydrodipicolinate synthase (*dapA_2*)	WP_039651239.1	909
*C. jejuni*	Hypothetical protein **	SUW97209.1	552
*C. jejuni*	Hypothetical protein **	MBX0540796.1	183
*C. jejuni*	Methyl-accepting chemotaxis protein **	WP_257408974.1	420
*C. jejuni* subsp. *doylei*	Hypothetical protein	ABS44036.1	231
*C. jejuni* subsp. *jejuni*	Hypothetical protein	ADT66919.1	378
*C. lari*	Major outer membrane protein (*porA_2*)	WP_317728191.1	525
*C. lari* subp. *lari*	Hypothetical protein	EFN2797389.1	1575
*C. lari* subsp. *concheus*	Hypothetical protein **	EAJ5700938.1	1353
*C. rectus*	Hypothetical protein **	WP_002945817.1	225
*C. upsaliensis*	Spermidine export protein (*mdlj*) **	WP_176318412.1	330
*C. upsaliensis*	Hypothetical protein **	WP_176318076.1	162
*C. upsaliensis*	Hypothetical protein	WP_004277133.1	402
*C. upsaliensis*	Hypothetical protein	EAJ0411667.1	666
*C. ureolyticus*	Hypothetical protein **	WP_202751539.1	891
*C. volucris*	Peptidoglycan O-acetyltransferase (*patA*)	WP_149104689.1	1500
*C. volucris*	Methyl-accepting chemotaxis protein (*mcpA*)	QEL09028.1	1977

** These targets are highly specific for those organisms.

## Data Availability

All whole genome sequences of isolates used for this study were downloaded from the National Center for Biotechnology Information (NCBI); see Supplementary Materials for all accession numbers.

## Supplementary Materials

The following supporting information can be downloaded at https://www.mdpi.com/article/10.3390/pathogens14050477/s1, Table S1: Information on specific genomic targets of the pathogenic species and subspecies.
